# Effects of imidacloprid-induced hormesis on the development and reproduction of the rose-grain aphid *Metopolophium dirhodum* (Hemiptera: Aphididae)

**DOI:** 10.3389/fphys.2023.1113464

**Published:** 2023-02-03

**Authors:** Xinan Li, Yaping Li, Xun Zhu, Xiangrui Li, Dengfa Cheng, Yunhui Zhang

**Affiliations:** ^1^ State Key Laboratory for Biology of Plant Disease and Insect Pests, Institute of Plant Protection, Chinese Academy of Agricultural Sciences, Beijing, China; ^2^ School of Resource and Environmental Sciences, Henan Institute of Science and Technology, Xinxiang, China; ^3^ Scientific Observing and Experimental Station of Crop Pests in Guilin, Ministry of Agriculture, Guilin, China

**Keywords:** hormesis, imidacloprid, *Metopolophium dirhodum*, longevity, fecundity

## Abstract

Field populations of insect pests are affected by sub-lethal doses of insecticides, leading to hormesis. Imidacloprid is a neonicotinoid insecticide widely used to control various sucking insect pests, including aphids. In this study, the effects of sub-lethal concentrations of imidacloprid on the life table traits of the rose-grain aphid *Metopolophium dirhodum* (Walker) were evaluated on parental and first filial generations. The results showed that sub-lethal concentrations of imidacloprid significantly reduced the fecundity, adult longevity, and reproductive period of *M. dirhodum* in parental generation (F_0_). However, the imidacloprid-induced hormetic effects on development and reproduction were detected in the F_1_ generation. These hormetic effects were indicated by significantly higher adult longevity, fecundity, survival rate, intrinsic and finite rates of increase, and net reproductive rate of first filial generation (F_1_) of *M. dirhodum*. Our finding indicated that the application of sub-lethal concentrations of imidacloprid inhibited parental generation (F_0_), but it significantly stimulated the population growth of filial generation (F_1_) in the *M. dirhodum*. The results support the inclusion of insecticides in integrated pest management programs for managing wheat aphids.

## Introduction

The insecticide concentration initially applied for managing insect pests degrades with the passage of time (Desneux and Fauvergue, 2005). The insect pests are eventually exposed to these time driven sub-lethal insecticide concentrations (Desneux et al., 2007). This exposure may affect their current and future generations. These sub-lethal concentrations induce physiological and behavioral changes in individuals that survive the initial pesticide exposure (Desneux et al., 2007). In some insects, sub-lethal pesticide exposure adversely affect survival, longevity, fecundity, developmental time, neurophysiological processes, biochemistry, immune capacity (Guo et al., 2013; Ceuppens et al., 2015; Ma et al., 2022), and even induce multiple behaviours (Samuelson et al., 2016). Sub-lethal insecticidal doses may also increase pest infestation by stimulating the growth and reproduction of arthropods (Cordeiro et al., 2013), including green peach aphid *Myzus persicae* (Tang et al., 2015; Tang et al., 2019), English grain aphid *Sitobion avenae*, bird cherry-oat aphid *Rhopalosiphum padi* (Xin et al., 2019), and cotton aphid *Aphis gossypii* (Ullah et al., 2020).

The biphasic dose-responsive adaptive response (i.e., hormesis) is characterized by low-dose stimulations and high-dose inhibitions (Guedes and Cutler, 2014). Hormetic responses have been detected in various organisms, including insects exposed to insecticides (Calabrese and Baldwin, 2001). The general overcompensation for a disruption in homeostasis (e.g., toxicity) is one of the mechanisms underlying hormesis (Guedes and Cutler, 2014). Additionally, hormesis is an essential consideration when evaluating the impact of pesticides in insect pest management strategies as it may also lead to pest resurgence (Shi et al., 2011; Chen et al., 2015).

Wheat aphids are one of the most harmful insect pests, which adversely affects grain crop production, and are responsible for considerable economic losses (Chopa and Descamps, 2012). The rose-grain aphid, *Metopolophium dirhodum* Walker (Hemiptera: Aphididae) is a major wheat aphid species that substantially lowers the productivity of cultivated winter cereals (Cannon, 1986; Ma et al., 2003). It was first detected in the 1970s in crops cultivated in South America. Since then, it has spread to new areas worldwide as an important pest of wheat (Chopa and Descamps, 2012; Abdelaziz et al., 2018; Honek et al., 2018). The nymphs and adults of this aphid, feed on phloem fluids from wheat plants during the wheat seedling and jointing stages. Aphid feeding exacerbates nutrient deficiency in wheat plant, resulting in the low grain production. Additionally, the aphids secrete honeydew, which can cover the leaf surface, and then hinders plant respiration and photosynthesis, ultimately leading to low-quality wheat and yield losses up to 27%–30% (Cannon, 1986). Also, the rose-grain aphid may function as a vector for several plant viruses that can damage cereal crops, especially the barley yellow dwarf virus (*Luteovirus*) (Chopa and Descamps, 2012).

Synthetic insecticides have a key role in modern pest management. Imidacloprid is the first commercially available systemic neonicotinoid insecticide. Its belongs to IRAC class 4A (nicotinic acetylcholine receptor (nAChR) competitive modulators) and blocks the nicotinic acetylcholine receptors in the central and peripheral nervous systems of insects (Palumbo et al., 2001; Byrne et al., 2003; Fernandez et al., 2009). Because of its long-lasting efficacy against diverse homopterous insect pests and relatively low toxicity to non-target organisms, imidacloprid has been commonly used to control insect pests such as *M. persicae*, *Bemisia tabaci* and *Nilaparvata lugens* (Palumbo et al., 2001; Byrne et al., 2003; Liu and Han, 2006). It has also been used in seed treatments for the long-term control of residual wheat aphids (Ahmed et al., 2001). Imidacloprid-induced pest resurgence, including those due to hormesis, have been reported for many insect pests such as the *M*. *persicae* (Yu et al., 2010) and *A*. *gossypii* (Ullah et al., 2019). However, the possible effects of sub-lethal doses of imidacloprid on *M. dirhodum* remain relatively unknown. In the present study, the sub-lethal effects of imidacloprid on *M. dirhodum* were investigated based on two-sex life tables, with a focus on transgenerational effects.

## Materials and methods

### Aphid rearing

The *M. dirhodum* population used in this research was sampled in 2016 from wheat fields located at the Pest Sciences Observation and Testing station in Haidian district, Beijing, China. After field collection, the aphids were maintained on young winter wheat (*Triticum aestivum* Linnaeus) plants in a growth chamber set at 18°C ± 1°C and 60% ± 10% relative humidity, with a 16-h light/8-h dark photoperiod. The aphids were not exposed to any insecticides prior to this study.

### Imidacloprid application

Imidacloprid (Gaucho® 600FS) (97.4%, technical grade) was obtained from Bayer Crop Science (Beijing, China). A stock solution was prepared in acetone (Beijing Chemical Works, China) and diluted with water containing 0.05% (w/v) Triton X-100 (Beijing Solarbio Science and Technology Co., Ltd., China) to concentrations appropriate for generating dose-response curves between 0% and 100% *M. dirhodum* mortality.

### Bioassay

A wheat seedling dipping method was used in this study (Gong et al., 2020). Briefly, the imidacloprid stock solution was diluted with distilled water containing 0.05% (w/v) Triton-X to generate nine treatment solutions (i.e., 12.5, 25, 50, 200, 600, 1,200, 1,500, 1,800, and 2,000 mg/L). Wheat seedlings were dipped in the prepared imidacloprid solutions or distilled water containing 0.05% (w/v) Triton-X (control) for 10 s. The seedling roots were wrapped with moistened cotton. The seedlings were then air-dried at room temperature and then placed in 500-mL plastic plates, each containing approximately 10 *M. dirhodum* adults. The treated and control insects were incubated in a plant growth chamber maintained at 18°C ± 1 °C and 60% ± 10% relative humidity, with a 16-h light/8-h dark photoperiod. Insect mortality was recorded after 24 h. If no more than two legs moved in response to a slight touch with a soft brush, the individual was considered dead. The imidacloprid treatments along with the control treatment were replicated ten times.

### Life table analysis

A life table study was completed with 50, 100, and 200 mg/L imidacloprid solutions (prepared in distilled water) and water containing 0.05% (w/v) Triton X-100 as the control. Wheat seedlings were dipped in the imidacloprid or control solutions for 10 s. A cohort of 90 third instar nymphs was treated with imidacloprid as described in bioassay section. After 24 h, the surviving aphids were individually transferred to pots (10 × 10 × 10 cm) containing young winter wheat plants. The short-term (i.e., in the parental generation) sub-lethal effects of imidacloprid on the treated *M. dirhodum* (F_0_) fecundity and survival were determined. The number of new born nymphs was recorded and the offspring were removed daily until death. The long-term (i.e., in the filial generation) sub-lethal effects of imidacloprid on *M. dirhodum* (F_1_) life table parameters were also examined, including the developmental period, life span, survival rate and fecundity. Accordingly, the life table data of the filial generation were recorded. For each treated female, one or two new born nymphs produced overnight were transferred to a new wheat plant, i.e., 100–120 newborn nymphs (≤24-h old) were observed individually in each group. The number of new-born nymphs was recorded and the offspring were removed daily until death. In each experiment, the aphids were transferred to new plants every 3–4 days.

### Data analyses

Corrected aphid mortality was calculated using Abbott’s formula. The LC_15_, LC_25_, LC_35_, were calculated with 95% confidence interval (95% CI), and slope were calculated by a probit analysis using the DPS software (version 7.05). The raw life history data for the *M. dirhodum* F_0_ and F_1_ were evaluated with the TWOSEX-MSChart program (Chi, 2022b), which is based on an age-stage, two-sex life table (Chi, 1988). A bootstrap test with a sample size of 100,000 was completed to detect differences among the means and standard errors of the populations and minimize the variation in the results (Efron and Tibshirani, 1993). To estimate the total population growth, the analysis of the initial *M. dirhodum* population (50 newborn nymphs) was projected to 60 days based on the above data with the TIMING-MSChart program (Chi, 2022a).

## Results

### The sub-lethal concentrations of imidacloprid against *M. dirhodum*

The toxicity of imidacloprid to the *M. dirhodum* adults was determined, the results show that the estimated LC_15_, LC_25_, LC_35_, and LC_50_ values were 53.936 mg/L (95% CI: 22.867–94.934 mg/L), 114.734 mg/L (95% CI: 59.716–182.705 mg/L), 209.699 mg/L (95% CI: 124.736–317.737 mg/L), and 380 mg/L (95% CI: 274–852 mg/L), respectively. Finally, 50, 100, and 200 mg/L imidacloprid were used as the sub-lethal concentrations (i.e., LC_15_, LC_25_, and LC_35_, respectively) in subsequent experiments. The effects of these concentrations on third instar nymphs were evaluated. At 24 h after the 50, 100, and 200 mg/L imidacloprid treatments, the aphid mortality rates were 15% ± 0.925%, 24% ± 0.845%, and 35% ± 1.352%, respectively.

### The sub-lethal effects of imidacloprid on *M. dirhodum* parental generation (F_0_)

**FIGURE 1:**
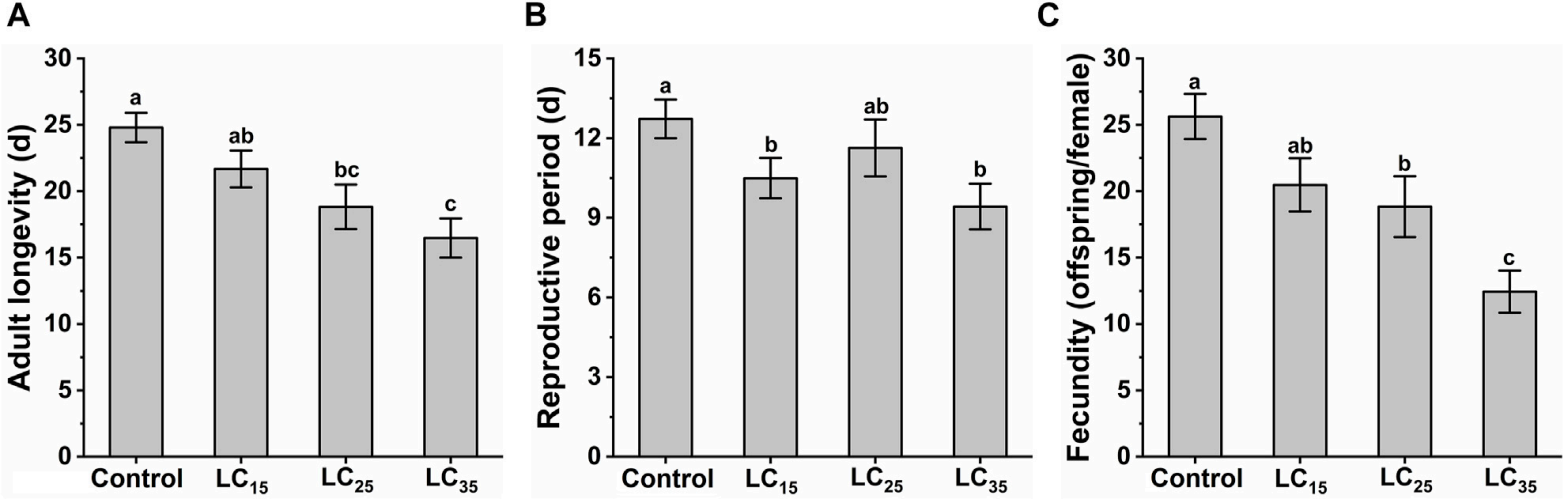
The adult longevity **(A)**, reproductive period **(B)** and fecundity **(C)** of *Metopolophium dirhodum* F_0_ generation under control conditions, treated with 50 mg/L of imidacloprid (LC_15_), 100 mg/L of imidacloprid (LC_25_) and 200 mg/L of imidacloprid (LC_35_).

The *M*. *dirhodum* third instar nymphs were treated with three sub-lethal concentrations of imidacloprid (50, 100, and 200 mg/L). The effects of different sub-lethal doses of imidacloprid on adult longevity, reproductive period and fecundity of *M. dirhodum* F_0_ are shown in Figure 1. The results show that the sub-lethal dose of imidacloprid had a significant negative effect on the adult longevity, reproductive period and fecundity of *M. dirhodum* F_0_, which showed a trend of significant decrease with the increase of the concentrations of imidacloprid.

### Transgenerational sub-lethal effects of imidacloprid on the developmental duration, longevity and fecundity of the first filial generation (F_1_)

The developmental duration, longevity and fecundity for the *M. dirhodum* F_1_ whose parents were treated with three sub-lethal concentrations of imidacloprid are presented in Table 1. The filial *M. dirhodum* aphids (first to fourth instars) developed rapidly following all treatments. Moreover, except for the third instar nymphs, the development was significantly negatively (first instars nymphs) and positively (second and fourth instar nymphs) affected by the three sub-lethal concentrations of imidacloprid. The pre-adult stage duration of F_1_ individuals tended to decrease, which was in contrast to the increase in the average adult stage duration following the imidacloprid treatments (relative to the effects of the control solution). The adult and total longevity of the F_1_ individuals was highest in response to the 50 mg/L imidacloprid treatment (LC_15_). The adult pre-reproductive period (APRP) and total pre-reproductive period (TPRP) was significantly reduced by the imidacloprid treatments, but the reproductive period was not significantly different between imidacloprid and the control treatments. Also, the fecundity of the F_1_ individuals was highest in response to the 100 mg/L imidacloprid treatment (LC_25_).

**TABLE 1:** The sub-lethal effects of imidacloprid on developmental duration and fecundity of the F_1_ generation of *Metopolophium dirhodum*.

Parameter^a^	Control	Imidacloprid leaf treatment (Mean ± SE)		
		LC_15_	LC_25_	LC_35_
N1 (d)	1.58 ± 0.10 c	2.05 ± 0.06 b	2.05 ± 0.06 b	2.40 ± 0.06 a
N2 (d)	2.25 ± 0.10 a	1.86 ± 0.06 b	1.81 ± 0.06 b	1.72 ± 0.06 b
N3 (d)	1.95 ± 0.12 a	1.96 ± 0.08 a	1.77 ± 0.08 a	1.88 ± 0.07 a
N4 (d)	3.05 ± 0.13 a	2.54 ± 0.09 b	2.67 ± 0.09 b	2.52 ± 0.08 b
Pre-adult (d)	8.82 ± 0.20 a	8.41 ± 0.11 ab	8.29 ± 0.11 b	8.52 ± 0.10 ab
Adult longevity (d)	31.27 ± 0.75 c	35.42 ± 0.61 a	33.47 ± 0.56 b	32.47 ± 0.59 bc
Total longevity (d)	40.09 ± 0.73 b	43.51 ± 0.66 a	41.07 ± 0.72 b	40.99 ± 0.57 b
APRP (d)	1.55 ± 0.12 a	1.23 ± 0.12 ab	1.02 ± 0.08 bc	0.88 ± 0.07 c
TPRP (d)	10.37 ± 0.23 a	9.64 ± 0.17 b	9.31 ± 0.13 b	9.40 ± 0.10 b
Reproductive period (d)	17.35 ± 0.78 a	17.32 ± 0.59 a	18.66 ± 0.58 a	17.62 ± 0.56 a
Fecundity (nymphs per female)	40.39 ± 2.44 b	42.39 ± 1.95 ab	47.60 ± 2.05 a	45.68 ± 2.01 ab

^a^ N1, first nymph stage; N2, second nymph stage; N3, third nymph stage; N4, fourth nymph stage; Pre-adult, complete nymph stage; APRP, adult pre-reproductive period; TPRP, total pre-reproductive period. Data in the table are represented as mean ± SE, estimated with bootstrapping (100,000). Different letters in the same row indicated significantly different (*p* < 0.05) by the paired bootstrap test.

### Transgenerational sub-lethal effects of the imidacloprid on survival rates, life expectancy and reproductive value of the *M. dirhodum* F_1_ generation

**FIGURE 2:**
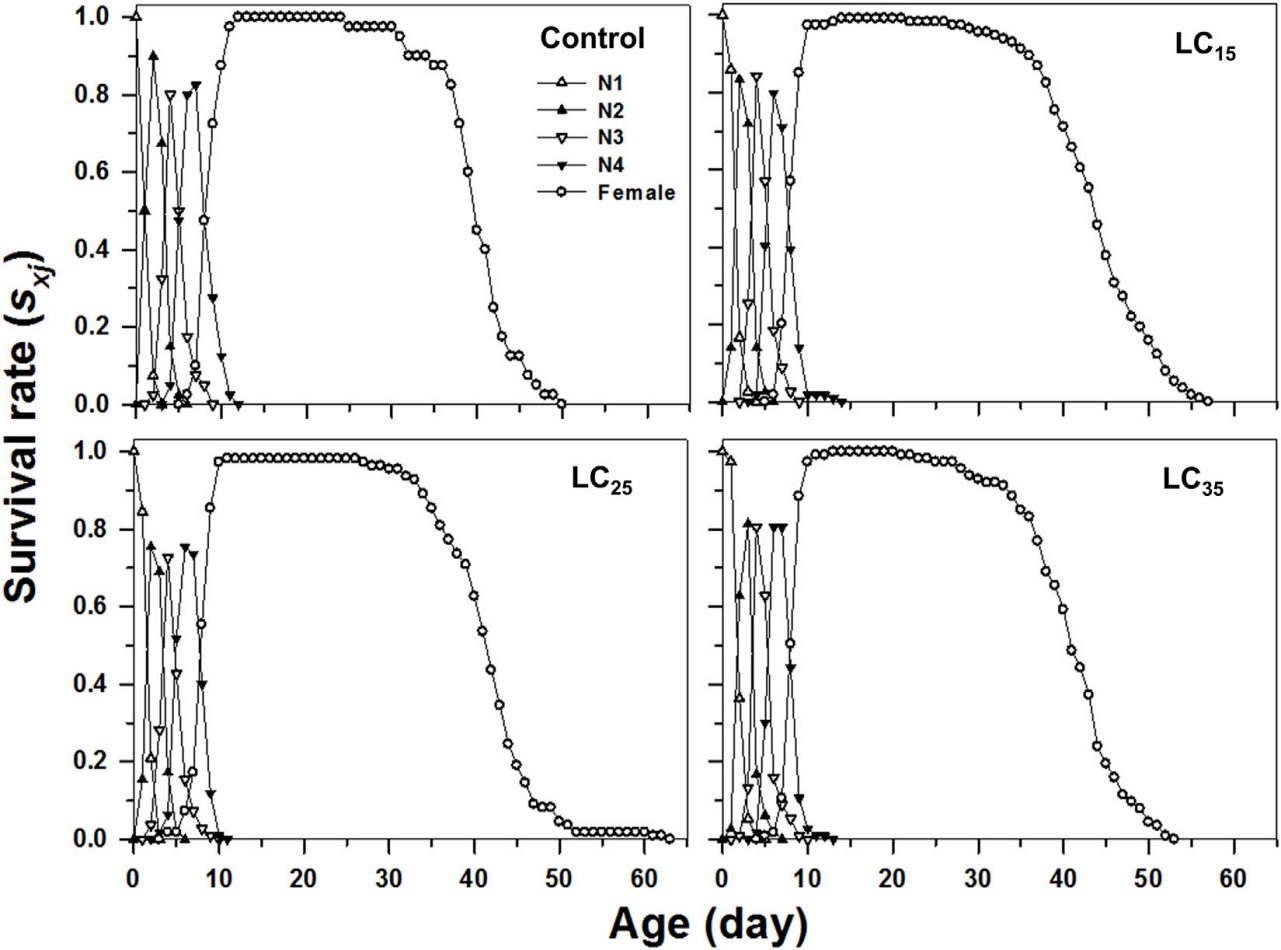
Age-stage-specifc survival rates (*s*_*xj*_) of *Metopolophium dirhodum* individuals of the F_1_ generation under control conditions, treated with 50 mg/L of imidacloprid (LC_15_), 100 mg/L of imidacloprid (LC_25_) and 200 mg/L of imidacloprid (LC_35_).

**FIGURE 3:**
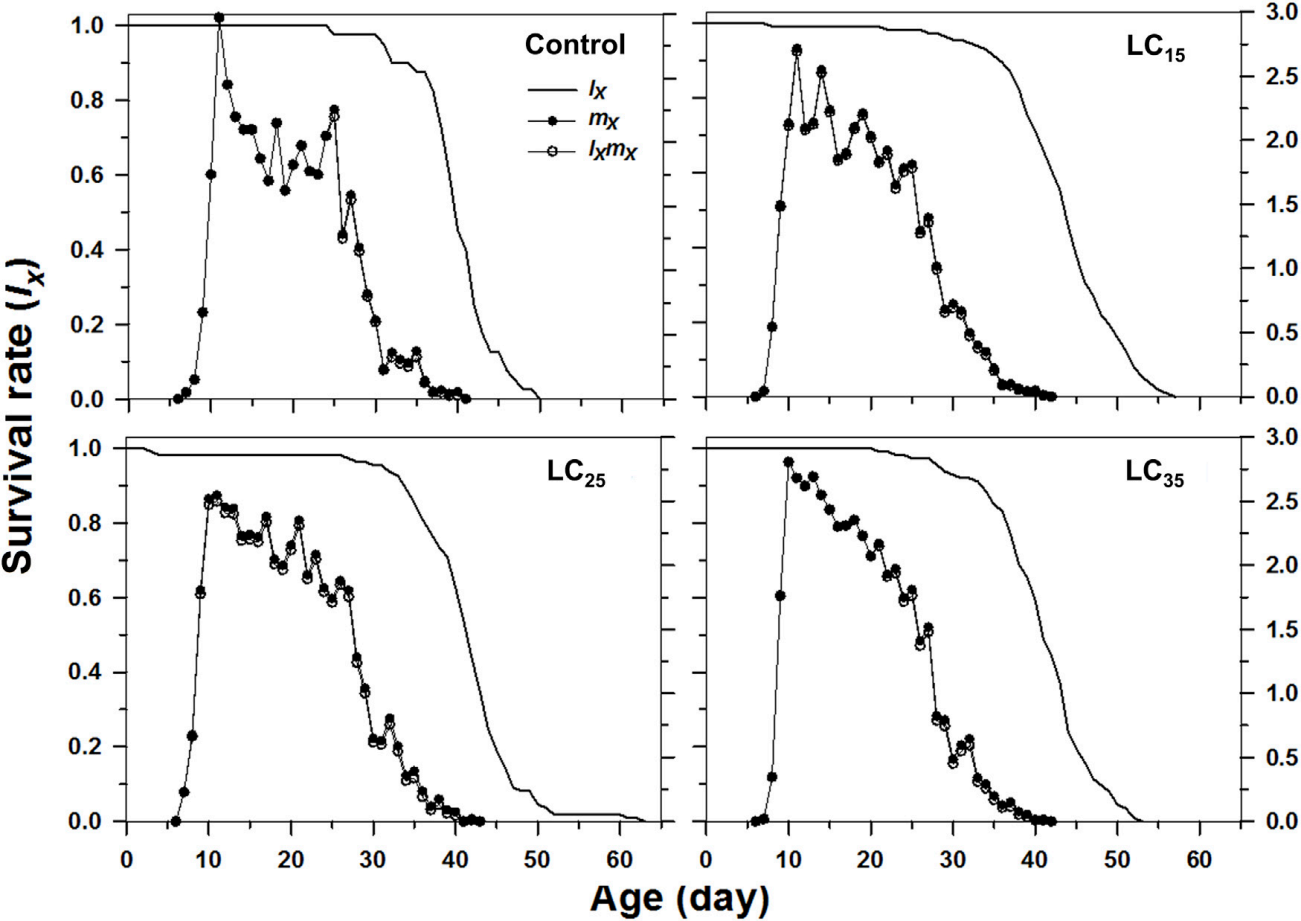
Age-specifc survival rates (*l*_*x*_), age-specifc fecundity (*m*_*x*_), and net maternity (*l*_*x*_ *m*_*x*_) of *Metopolophium dirhodum* individuals of the F1 generation under control conditions, treated with 50 mg/L of imidacloprid (LC_15_), 100 mg/L of imidacloprid (LC_25_) and 200 mg/L of imidacloprid (LC_35_).

The curves of age-stage specific survival rates (*s*_*xj*_) reflect the high age-stage-specific survival rates for the females following all treatments, however, the imidacloprid-treated *M. dirhodum* F_1_ generation had a higher survival rate than that of the control in later stages (Figure 2). The age-specific survival rate (*l*_*x*_), the age-specific fecundity (*m*_*x*_), and the age-specific maternity (*l*_*x*_ *m*_*x*_) reflected the effects of the increasing imidacloprid concentrations on the *M. dirhodum* population over time, which indicate that these parameters were higher in different doses of imidacloprid treated groups of aphids middle and later stage (Figure 3).

**FIGURE 4:**
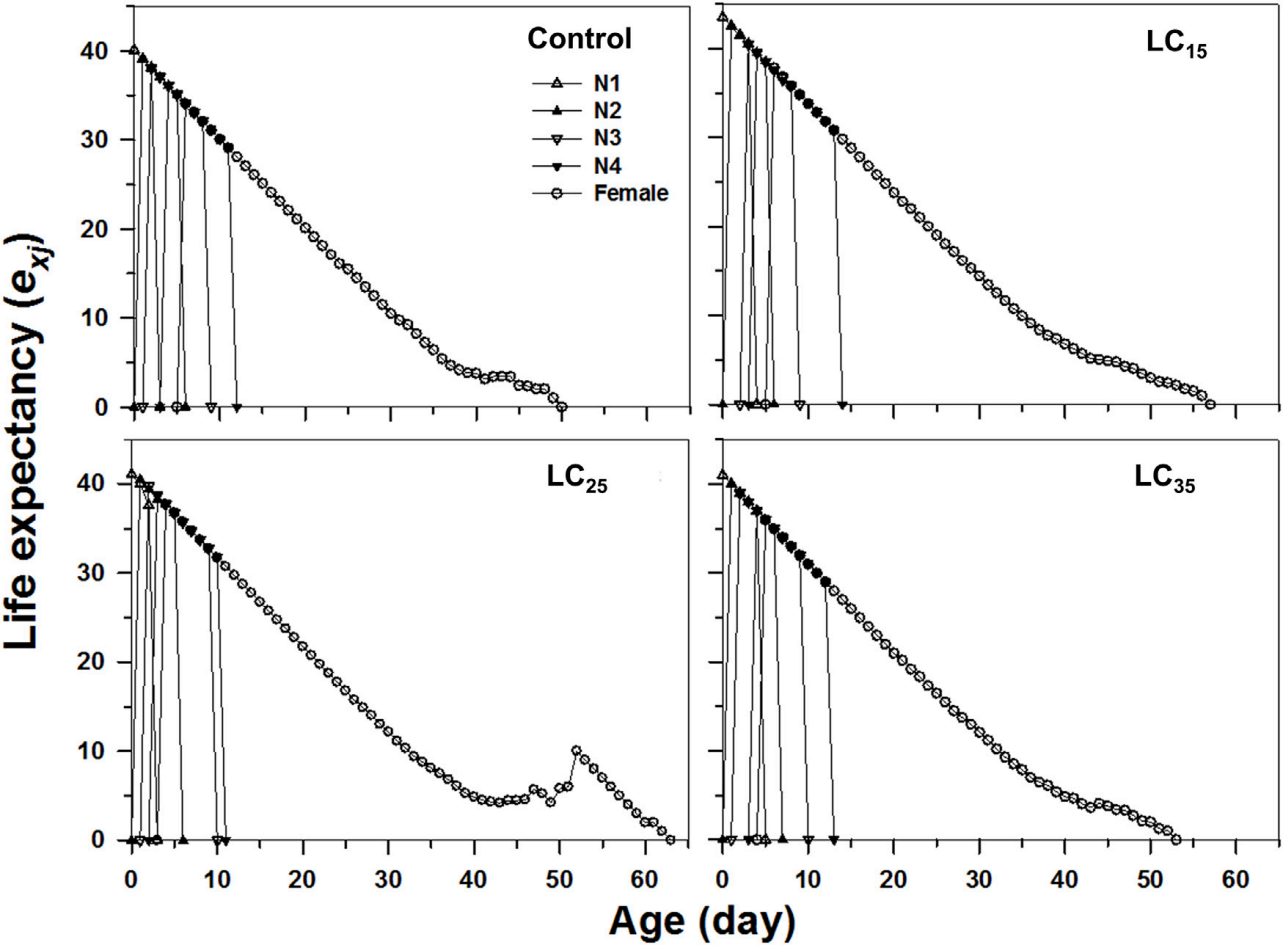
Age-stage-specifc life expectancy (*e*_*xj*_) of *Metopolophium dirhodum* individuals of the F1 generation under control conditions, treated with 50 mg/L of imidacloprid (LC_15_), 100 mg/L of imidacloprid (LC_25_) and 200 mg/L of imidacloprid (LC_35_).

**FIGURE 5:**
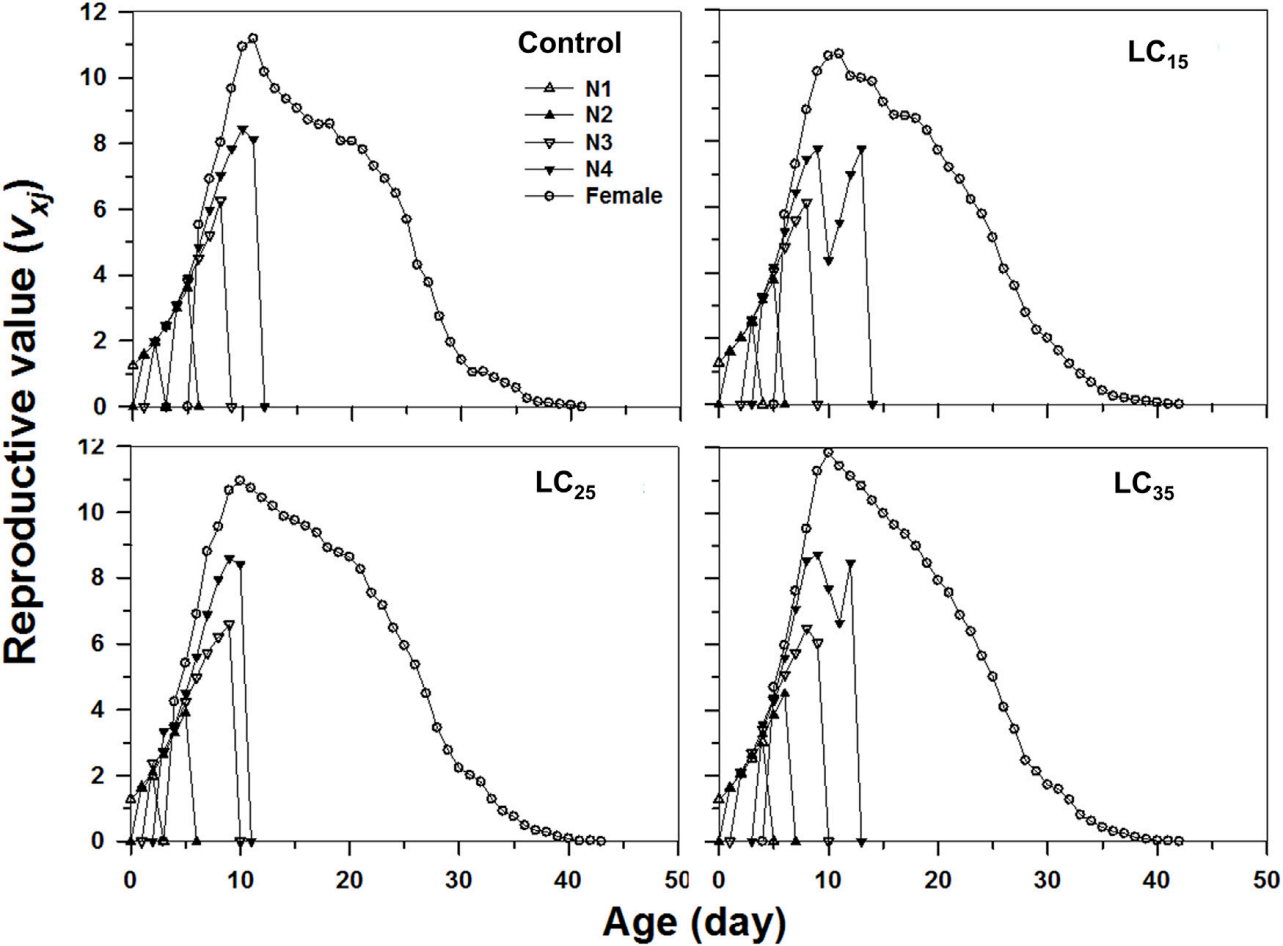
Age-stage-specifc reproductive value (*v*_*xj*_) of *Metopolophium dirhodum* individuals of the F1 generation under control conditions, treated with 50 mg/L of imidacloprid (LC_15_), 100 mg/L of imidacloprid (LC_25_) and 200 mg/L of imidacloprid (LC_35_).

The age-stage specific life expectancy (*e*_*xj*_) refers to the predicted survival time of an individual at age *x* and stage *j*. Compared with control, the F_1_ individuals produced by F_0_ imidacloprid-treated had a higher life expectancy (Figure 4). Moreover, newborn *M. dirhodum* nymphs were expected to live for 57, 63, and 53 days following the 50, 100, and 200 mg/L imidacloprid treatments, respectively, for only 50 days in response to the control treatment (Figure 4). An analysis of the *M. dirhodum* age-stage-specific reproductive rate (*v*_*xj*_) following each imidacloprid treatment revealed that it was highest for LC_35_ (day 10) than for the control (day 12), as well as for LC_15_ and LC_25_ (day 11) (Figure 5).

### The effects of the imidacloprid on population parameters and total population size of the *M. dirhodum* F_1_ generation

Population life-table parameters of the control and imidacloprid-treated *M. dirhodum* F_1_ generation are listed in Table 2. The data presented in this table shows that the imidacloprid treatments decreased the mean generation time (*T*) of *M. dirhodum* F_1_ generation. However, increases in imidacloprid concentrations significantly increased the net reproductive rate (*R*_0_), intrinsic rate of increase (*r*) and finite rate of increase (*λ*), indicated that all imidacloprid treatments induced a rapid *M. dirhodum* population growth.

**TABLE 2:** Sub-lethal effects of imidacloprid on population parameters of the F_1_ generation of *Metopolophium dirhodum*.

Parameter^a^	Control	Imidacloprid leaf treatment (Mean ± SE)		
		LC_15_	LC_25_	LC_35_
Intrinsic rate of increase/*r*	0.2243 ± 0.0051 b	0.2347 ± 0.0036 ab	0.2433 ± 0.0041 a	0.2435 ± 0.0032 a
Finite rate of increase/*λ*	1.2514 ± 0.0064 b	1.2646 ± 0.0045 ab	1.2755 ± 0.0052 a	1.2757 ± 0.0041 a
Net reproductive rate/*R*_0_	40.40 ± 2.43 b	42.02 ± 1.97 ab	46.73 ± 2.10 a	45.68 ± 2.01 ab
Mean generation time/*T*	16.48 ± 0.25 a	15.92 ± 0.17 ab	15.80 ± 0.19 b	15.69 ± 0.14 b

^a^ Data in the table are represented as mean ± SE, estimated with bootstrapping (100,000). Different letters in the same row indicated significantly different (*p* < 0.05) by the paired bootstrap test.

**FIGURE 6:**
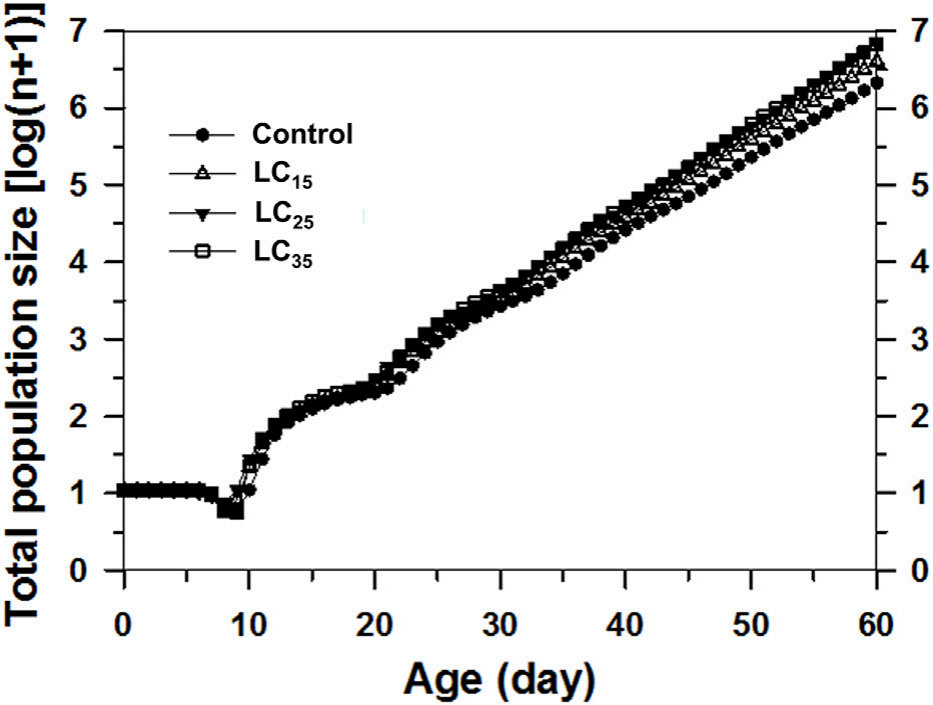
Comparison of population projections for *Metopolophium dirhodum* whose parental females treated with three concentrations of imidacloprid or as the control, based on the age-stage, two-sex life table. The regression formulas describe the linear population growth of each cohort from day 40 onwards as the population approached the stable age-stage distribution.

The projected population was analysed based on the age-stage, two-sex life table and the data for F_1_ (Figure 6). The control population on day 60 was expected to reach approximately 2.47 million aphids. In contrast, the 50 (LC_15_), 100 (LC_25_), and 200 (LC_35_) mg/L imidacloprid treatments on day 60 were predicted to result in 4.72, 7.81, and 7.80 million aphids, respectively. Forty days later, the *M. dirhodum* population growth curves calculated on a logarithmic scale were nearly linear, implying that *M. dirhodum* populations were approaching the stable age-stage distribution. Such linear population increases are indicated by the slopes of the regression lines, which are equal to log(*λ*) for each cohort. These results suggest that an exposure to sub-lethal concentrations of imidacloprid induced *M. dirhodum* population growth.

## Discussion

Imidacloprid is a typical neonicotinoid insecticide widely used to control various sucking pests, including aphids (Palumbo et al., 2001; Byrne et al., 2003; Fernandez et al., 2009) Target and non-target arthropods are often exposed to low insecticide concentrations in treated fields. The potential sub-lethal effects of insecticides should be evaluated while developing effective integrated pest management programs (Desneux et al., 2007; Guedes et al., 2016). Assessment of sub-lethal insecticidal effects on target insects is critical for enhancing the pesticide efficiency (Liang et al., 2012; Xiao et al., 2015). The primary objective of the current study was to clarify the sub-lethal effects of imidacloprid on the development of the *M*. *dirhodum* population.

Insecticide-induced hormesis may result in pest resurgence and/or secondary pest outbreaks, which may necessitate additional pesticide applications and a steady accumulation of potentially harmful chemicals in the field (Cordeiro et al., 2013; Guedes et al., 2016). Hormesis due to insecticide applications has been observed in other aphid species, including *M. persicae* treated with low imidacloprid concentrations (Christopher Cutler et al., 2009; Murali-Mohan et al., 2013) and *A. gossypii* exposed to sulfoxaflor, pirimicarb, and flonicamid (Koo et al., 2015; Xiao et al., 2015; Chen et al., 2016).

Earlier investigations indicated that treatments with sub-lethal doses of thiamethoxam adversely affect *Hippodamia variegate* and *Bradysia odoriphaga* population growth (Rahmani and Bandani, 2013; Zhang et al., 2014). In the current study, the sub-lethal dose of imidacloprid had also a significant adverse effect on *M*. *dirhodum* F_0_ generation, indicating that the stimulatory (hormetic) effects were not observed in F_0_ individuals treated with low concentrations of imidacloprid. This is consistent with the reported lack of hormetic effects in the F_0_ generations of *A. gossypii* exposed to a sub-lethal concentration of afidopyropen and sulfoxaflor (Chen et al., 2016; Ma et al., 2022) and an imidacloprid-resistant *A. gossypii* strain treated with a low lethal concentration of imidacloprid (Shi et al., 2011). Similar effects were also observed in *M. persicae*, *B. brassicae*, *B. tabaci*, and *Apolygus lucorum* (Meyer-Dür) treated with sub-lethal doses of imidacloprid (Lashkari et al., 2007; Wang et al., 2008; Tan et al., 2012; He et al., 2013).

In this study, assessing the transgenerational effects of imidacloprid treatments on *M. dirhodum*, we observed that an exposure to sub-lethal concentrations of imidacloprid in the F_0_ generation (i.e., parent generation) significantly stimulated the population growth of the F_1_ generation, the analysed life table parameters (*r*, *λ*, and *R*_**0**_), fecundity, longevity, and survival of the F_1_ generation were positively affected by the sub-lethal concentrations of imidacloprid treatments. Additionally, the population prediction results show that an exposure to sub-lethal concentrations of imidacloprid induced *M*. *dirhodum* population growth. Similar effects on population growth were observed in an earlier study on sweetpotato whitefly *B*. *tabaci*, which proved that imidacloprid increases the gross reproduction rate, but does not significantly affect the mean generation time (Esmaeily et al., 2014). Another study involving field experiments in Australian revealed that the egg production and population development of the Australian predatory mite *Amblyseius victoriensis* (Womersley) significantly increase in response to systemic spray treatments of imidacloprid (James, 1997). Other investigations confirmed that a low dose of imidacloprid shortens the mean generation time of cabbage aphid *Brevicoryne brassicae* (Lashkari et al., 2007), whereas a sub-lethal imidacloprid concentration significantly extends the mean generation time of *B. tabaci*, while also increasing fecundity and egg production (Sohrabi et al., 2011). Furthermore, the systemic application of imidacloprid to control psyllids on pear trees reportedly increase the fecundity of mite populations (James and Price, 2002).

The stimulated reproduction of insects exposed to low lethal concentration of insecticides is due to hormesis (Guedes and Cutler, 2014). The imidacloprid or one of its metabolites might have altered aphid physiology, with potential consequences for reproduction and population growth. There are reports describing the increased fecundity in *Tetranychus urticae*, *A. victoriensis*, and *Tryporyza incertulas* due to low imidacloprid concentrations (James and Price, 2002; Wang et al., 2005). Additionally, the exposure of *M. persicae* to sub-lethal doses of imidacloprid was observed to result in a hormetic effect on fecundity in the F_2_ generation (Christopher Cutler et al., 2009). In this study, the hormetic effects of imidacloprid on the life table parameters of *M. dirhodum* were detected in F_1_ generation, and fecundity was highest for LC_25_ and the adult longevity was longest for LC_15_. When the insecticide concentration is too low, the overcorrection is not triggered or it is not discernible (Stebbing, 2003). These results imply that the development of hormetic effects may be influenced by time and the imidacloprid concentration, and the complex mechanism underlying the imidacloprid dose effect on the occurrence of hormesis.

In conclusion, our findings indicated that the application of sub-lethal concentrations of imidacloprid has inhibitory effects on the parental generation (F_0_), but has stimulatory effects on the first filial generation (F_1_). Considering all of the arthropod biological processes affected by pesticides, it is possible that hormetic responses are induced in arthropods exposed to these chemicals (Kendig et al., 2010; Liang et al., 2012). Future research should examine the effects of various low lethal and sub-lethal insecticide concentrations to comprehensively characterize the putative hormetic responses of wheat aphid pests to neonicotinoid insecticides. The results of this research may be relevant for optimizing integrated pest management strategies involving neonicotinoid insecticides.

## Data Availability

The original contributions presented in the study are included in the article/supplementary material, further inquiries can be directed to the corresponding author.
