# Corrigendum: Effects of imidacloprid-induced hormesis on the development and reproduction of the rose-grain aphid *Metopolophium dirhodum* (Hemiptera: aphididae)

**DOI:** 10.3389/fphys.2023.1186884

**Published:** 2023-06-06

**Authors:** Xinan Li, Yaping Li, Xun Zhu, Xiangrui Li, Dengfa Cheng, Yunhui Zhang

**Affiliations:** ^1^ State Key Laboratory for Biology of Plant Disease and Insect Pests, Institute of Plant Protection, Chinese Academy of Agricultural Sciences, Beijing, China; ^2^ School of Resource and Environmental Sciences, Henan Institute of Science and Technology, Xinxiang, China; ^3^ Scientific Observing and Experimental Station of Crop Pests in Guilin, Ministry of Agriculture, Guilin, China

**Keywords:** hormesis, imidacloprid, *Metopolophium dirhodum*, longevity, fecundity

In the published article, there was an error in the note for Table 1 as published. The word “paired” was missing from the following sentence: “Different letters in the same row indicated significantly different (*p* < 0.05) by the bootstrap test.” The corrected table note appears below.

**TABLE 1:** The sub-lethal effects of imidacloprid on developmental duration and fecundity of the F_1_ generation of *Metopolophium dirhodum*.

Parameter^a^	Control	Imidacloprid leaf treatment (Mean ± SE)		
		LC_15_	LC_25_	LC_35_
N1 (d)	1.58 ± 0.10 c	2.05 ± 0.06 b	2.05 ± 0.06 b	2.40 ± 0.06 a
N2 (d)	2.25 ± 0.10 a	1.86 ± 0.06 b	1.81 ± 0.06 b	1.72 ± 0.06 b
N3 (d)	1.95 ± 0.12 a	1.96 ± 0.08 a	1.77 ± 0.08 a	1.88 ± 0.07 a
N4 (d)	3.05 ± 0.13 a	2.54 ± 0.09 b	2.67 ± 0.09 b	2.52 ± 0.08 b
Pre-adult (d)	8.82 ± 0.20 a	8.41 ± 0.11 ab	8.29 ± 0.11 b	8.52 ± 0.10 ab
Adult longevity (d)	31.27 ± 0.75 c	35.42 ± 0.61 a	33.47 ± 0.56 b	32.47 ± 0.59 bc
Total longevity (d)	40.09 ± 0.73 b	43.51 ± 0.66 a	41.07 ± 0.72 b	40.99 ± 0.57 b
APRP (d)	1.55 ± 0.12 a	1.23 ± 0.12 ab	1.02 ± 0.08 bc	0.88 ± 0.07 c
TPRP (d)	10.37 ± 0.23 a	9.64 ± 0.17 b	9.31 ± 0.13 b	9.40 ± 0.10 b
Reproductive period (d)	17.35 ± 0.78 a	17.32 ± 0.59 a	18.66 ± 0.58 a	17.62 ± 0.56 a
Fecundity (nymphs per female)	40.39 ± 2.44 b	42.39 ± 1.95 ab	47.60 ± 2.05 a	45.68 ± 2.01 ab

^a^ N1, first nymph stage; N2, second nymph stage; N3, third nymph stage; N4, fourth nymph stage; Pre-adult, complete nymph stage; APRP, adult pre-reproductive period; TPRP, total pre-reproductive period. Data in the table are represented as mean ± SE, estimated with bootstrapping (100,000). Different letters in the same row indicated significantly different (*p* < 0.05) by the paired bootstrap test.

In the published article, there was an error in the note for Table 2 as published. The word “paired” was missing from the following sentence: “Different letters in the same row indicated significantly different (*p* < 0.05) by the bootstrap test.” The corrected table note appears below.

**TABLE 2:** Sub-lethal effects of imidacloprid on population parameters of the F_1_ generation of *Metopolophium dirhodum*.

Parameter^a^	Control	Imidacloprid leaf treatment (Mean ± SE)		
		LC_15_	LC_25_	LC_35_
Intrinsic rate of increase/*r*	0.2243 ± 0.0051 b	0.2347 ± 0.0036 ab	0.2433 ± 0.0041 a	0.2435 ± 0.0032 a
Finite rate of increase/*λ*	1.2514 ± 0.0064 b	1.2646 ± 0.0045 ab	1.2755 ± 0.0052 a	1.2757 ± 0.0041 a
Net reproductive rate/*R*_0_	40.40 ± 2.43 b	42.02 ± 1.97 ab	46.73 ± 2.10 a	45.68 ± 2.01 ab
Mean generation time/*T*	16.48 ± 0.25 a	15.92 ± 0.17 ab	15.80 ± 0.19 b	15.69 ± 0.14 b

^a^ Data in the table are represented as mean ± SE, estimated with bootstrapping (100,000). Different letters in the same row indicated significantly different (*p* < 0.05) by the paired bootstrap test.

In the published article, there was an error in Table 2 as published. Some of the data in Table 2 should be kept 4 digits behind the decimal point**.** The corrected Table 2 and its caption appear below.

In the published article, there was an error. Some corrections have been made to **Results**, *Transgenerational sub-lethal effects of the imidacloprid on survival rates, life expectancy and reproductive value of the M. dirhodum F_1_ generation*, paragraph two. This sentence previously stated:

“The age-stage specific life expectancy (*e*_*xj*_) refers to the predicted survival of an individual at age *x* and stage *j* at a later age x. Compared with control, the F_1_ individuals produced by F0 imidacloprid-treated had a higher life expectancy (Figure 4). Moreover, newborn *M*. *dirhodum* nymphs were expected to live for 56, 62, and 52 days following the 50, 100, and 200 mg/L imidacloprid treatments, respectively, for only 49 days in response to the control treatment (Figure 4).”

In the corrected sentence, “survival” was changed to “survival time” due to a missing word, “at a later age x” was deleted as these were superfluous words, and “F0” was corrected to “F_0_” since “0” should be subscripted. Additionally, “56, 62, and 52 days” was changed to “57, 63, and 53 days” and “49 days” was changed to “50 days”. The first day that the life expectancy value in Figure 4 is 0 should be added, so the life expectancy value for each treatment should be added by 1 day.

The corrected sentence appears below:

“The age-stage specific life expectancy (*e*_*xj*_) refers to the predicted survival time of an individual at age *x* and stage *j*. Compared with control, the F_1_ individuals produced by F_0_ imidacloprid-treated had a higher life expectancy (Figure 4). Moreover, newborn *M*. *dirhodum* nymphs were expected to live for 57, 63, and 53 days following the 50, 100, and 200 mg/L imidacloprid treatments, respectively, for only 50 days in response to the control treatment (Figure 4).”

The authors apologize for these errors and state that this does not change the scientific conclusions of the article in any way. The original article has been updated.

